# Potential role of saffron and its components on miRNA levels in various disorders, a comprehensive review

**DOI:** 10.22038/IJBMS.2023.71915.15627

**Published:** 2023

**Authors:** Mohammad Reza Aslani, Farshad Armin, Ali Abedi, Elham Keramati, Hassan Ghobadi

**Affiliations:** 1 Lung Diseases Research Center, Ardabil University of Medical Sciences, Ardabil, Iran; 2 Department of Physiology, Faculty of Medicine, Ardabil University of Medical Sciences, Ardabil, Iran

**Keywords:** Crocetin, Crocin, miRNAs, Saffron, Safranal

## Abstract

The potential therapeutic benefits of saffron and its active constituents have been investigated for the treatment of numerous illnesses. In this review, the impacts of saffron and its essential components on the levels of microRNAs (miRNAs) in different diseases have been delineated. Relevant articles were obtained through databases such as PubMed, Web of Sciences, Scopus, and Google Scholar up to the end of November 2022. miRNA expression has been altered by saffron and its active substances (crocin, crocetin, and safranal) which has been of great advantage in treating diseases such as cardiovascular, type 2 diabetes, cancers, gastrointestinal and liver disorders, central and peripheral nervous system disorders, asthma, osteoarthritis, ischemic-reperfusion induced injury conditions, and renal disorder. This study uncovered the potential restorative advantages of saffron and its derivatives, in miRNA imbalances in a variety of diseases.

## Introduction

***MicroRNAs***

**Figure 1:**
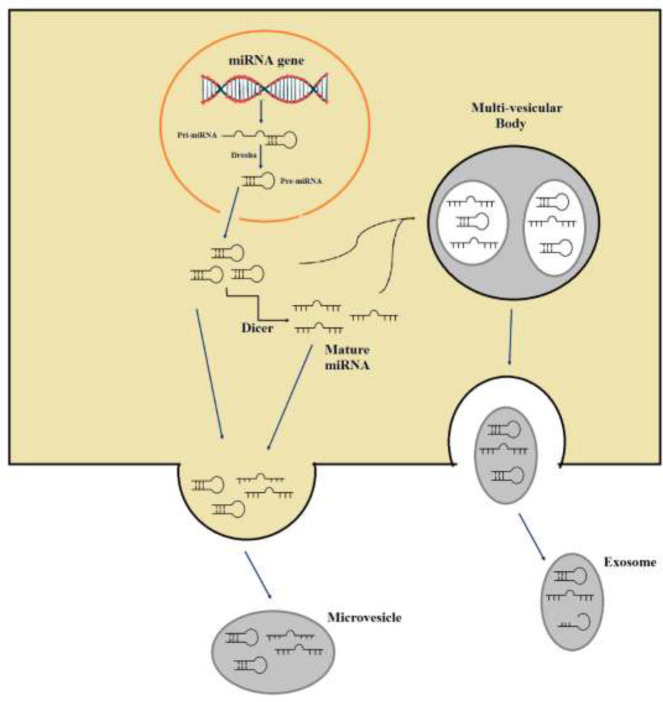
Biogenesis of miRNAs. pre-miRNA: precursor miRNAs, pri-miRNAs: primary miRNAs

MicroRNAs (miRNAs) consist of short, non-coding ribonucleic acids, typically having 22–25 nucleotides. Initially, primary miRNAs (pri-miRNAs) are constructed from DNA strands, which are then converted into precursor miRNAs (pre-miRNA) and mature miRNAs (1, 2). miRNAs and target mRNAs interact by utilizing the 3′ UTR, 5′ UTR, coding sequence, and gene promoters (1). The majority of research has shown that miRNAs are generally associated with the inhibition of gene expression, yet in some instances, they have been observed to activate gene expression (3). Evidence suggests that attaching to the 5’ UTR and coding regions usually reduces gene expression, while attaching to the promoter section usually leads to amplified gene expression (4). miRNA’s ability to regulate gene expression is dynamic and is referred to as a gene expression buffer (5). The formation of miRNAs is achieved via two distinct pathways. In the canonical pathway, Drosha (an RNase III enzyme) and the RNA binding protein DGCR8 convert the pri-miRNA into the pre-miRNA (6). However, proteins such as Drosha, Dicer, exportin 5, and Argonaute2 (AGO2) are utilized to complete the non-conventional miRNA biogenesis pathway (7) (Figure 1). 

Evidence suggests that relocating miRNAs to varying cellular sections can control cells’ activities such as translation and transcription (8). Apart from miRNA being found in various internal compartments, recent research has indicated their existence in external liquids like plasma, serum, saliva, cerebrospinal liquid, urine, breast milk, peritoneal fluid, colostrum, ovarian follicular liquid, bronchial lavage, tears, and seminal fluid. Research has shown extracellular miRNAs to be a viable indicator for different diseases. Remarkably, miRNAs can control autocrine, paracrine, and endocrine effects, resulting in the common conception that hormones are located in extracellular fluids (9, 10). MiRNAs can be identified in external fluids in the form of exosomes, apoptotic bodies, and microvesicles, or, alternatively, be linked to proteins including AGO2, high-density lipoprotein (HDL), and nucleophosmin 1 (NPM1) (11) (Figure 1). Research has proven that the emission of miRNAs to the external environment as a result of cell trauma or death can be a carefully orchestrated process in some circumstances. Exosomal miRNA release induces modulating effects on the development of target cells (12).

miRNAs are essential in mediating inflammatory and immune responses by modulating gene expression posttranscriptionally. As a result, miRNAs may be used as indicators of a variety of human diseases and therapy objectives for medical treatments (13). Numerous studies have documented the involvement of miRNAs in an array of medical conditions. An important factor in the development of diseases has been the dysfunction of miRNAs. Studies have revealed that alterations in miRNAs expression have had a significant impact on the severity of a range of diseases, including cardiovascular (14), cancers (15), gastrointestinal disorders (16), peripheral and central nervous system disorders (17), and metabolic diseases (18).

***Saffron and its ingredients***

As an expensive spice, saffron is made from the dried orange-red stigmas of *Crocus sativus* L., which is native to Iran. Since ancient times, this spice has been utilized to give flavor and color to food preparations (19). It has been revealed that saffron stigmas possess over 300 lipophilic and hydrophilic components, including vitamins, proteins, starches, carbohydrates, minerals, gum, mucilage, pigments like alpha and beta carotenes, picrocrocin, crocin, safranal, saponins, xanthone carotenoid, alkaloids, mangicrocin, aldehydes, and glycoside monoterpenes (20). Picrocrocin (apocarotenoid), safranal (monoterpene aldehyde), crocin, and crocetin (carotenoids derived from zeaxanthin) are the main active ingredients of saffron. Crocin, picrocrocin, and safranal are responsible for the yellow color, taste, and aroma of the spice (21). It has been demonstrated that saffron typically has 30% crocin, 5-15% picrocrocin, and almost 2.5% safranal (22). Gastrointestinal digestion can provide up to 50% availability of crocetin esters and 70% of picrocrocin, however, crocin is probably not obtainable through this procedure (23). Intestinal epithelial enzymes catalyze crocin to be changed into deglycosylated trans-crocetin, which then passes through the cellular membranes (24). 

Considering treating diseases with medicinal plants has been of great interest (25-29). For a significant time, saffron has been identified to contain therapeutic properties, and in addition to its traditional and general applications, it has been utilized in the treatment of several diseases. Recent investigations in the pharmaceutical field have demonstrated the effect of saffron and its active components in animal and human experiments, thus categorizing it as a medicinal plant (29, 30). Investigations have revealed the biological properties of saffron and its active constituents. Evidence has been provided that crocin has anti-inflammatory activity by suppressing cyclooxygenase-1 (COX1), cyclooxygenase-2 (COX2), prostaglandin-2 (PGE2), nuclear factor kappa B (NF-kB), inducible nitric oxide synthase (iNOS) and various inflammatory cytokines (IL- 6, TNF-α, IL-1β, IL-4, IL-5, IL17, and IL-33) (21, 22, 31). In addition, crocin and crocetin have been proven to be effective antioxidants by decreasing free radicals and lipid peroxidation and increasing reduced glutathione and antioxidant enzymes (superoxide dismutase (SOD), catalase (CAT), and glutathione peroxidase (GPx) (21, 29, 32, 33). Studies involving animals have illustrated that safranal has antioxidant, anti-inflammatory, and anti-apoptotic properties (21). Studies have demonstrated various mechanisms of saffron and its active ingredients on endoplasmic reticulum stress, smooth muscle relaxation, NF-kB, and JAC/STAT (34-37). Furthermore, a variety of human studies have revealed the beneficial effects of saffron and its derivatives in diseases including type 2 diabetes, cardiovascular disease, asthma, chronic obstructive pulmonary disease, polycystic ovary syndrome, depression, schizophrenia, anxiety, sarcopenia, Alzheimer’s disease, Parkinson’s disease, diabetic retinopathy, glaucoma, dyslipidemia, arteriosclerosis, hypertension, and renal failure (21, 29, 31, 33, 38). 

Considering that there needed to be a comprehensive study on the effects of saffron on miRNAs, the current study aimed to provide different studies focusing on the role of saffron on miRNAs.

***Method***

Investigating the influence of saffron and its active components on miRNA expression in various disorders was performed utilizing search databases such as PubMed, Web of Sciences, Scopus, and Google Scholar until the end of November 2022. Keywords such as (saffron, *Crocus sativus*), (crocin), (crocetin), (safranal), (picrocrocin), and (microRNA or miRNAs) were used for this review article. The current review included articles in English and Persian languages from renowned international journals. The search databases provided an accumulation of 174 articles, however after taking out the 105 duplicates, 69 remained for the study. Forty-four did not meet the inclusion criteria and finally, 25 articles were selected. The selected articles included human and animal studies related to cardiovascular diseases (39-44), cancer disorders (45-51), diabetes mellitus (52-55), nervous system disorders (56, 57), gastrointestinal disorders (58-61), asthma (37), and osteoarthritis (62).

## Results

***Cardiovascular diseases (CVD)***

*The effects of aqueous extract of saffron*

In various studies, a key role for miR-21 in the cardiovascular system has been defined, notably exhibiting a high level of expression in the cardiovascular system. Evidence exists of an unrestrained miR-21 expression in cardiovascular diseases (63). MiR-21 has a major influence on the activity of endothelial cells, resulting in the reduction of apoptosis and the reinforcement of these cells against oxidative stress (64). Elevated amounts of miR-21 were noted in individuals suffering from atherosclerosis, and its significant influence on the development of plaque has been exposed (65). On the other hand, miR-142-3p has been demonstrated to influence a range of cellular activities such as proliferation, apoptosis, and differentiation. Studies have indicated that the enhanced expression of miR-142-3p during hypertrophic development leads to heart failure through the initiation of increased apoptosis in an animal model (66).

In an *in vitro* study on human vascular endothelial cells (HUVECs) under conditions of oxidative stress induced by free radical generators, 2,2-azobis (2-amidinopropane) dihydrochloride (AAPH) and tertbutylhydroperoxide (TBHP), the effects of aqueous extract of saffron on the expression level of miR-21 and miR-142-3p have been evaluated. The mechanism of action for two stressors, TBHP and AAPH, in producing oxidative stress varies. TBHP causes oxidative stress in mitochondria, whereas AAPH induces oxidative stress through the cell membrane and the cytoplasm. Oxidative stress induced by AAPH and TBHP in HUVECs cells promoted an increase in miR-21 expression. Nevertheless, levels of miR-142-3p were only heightened under AAPH stress conditions. Treating HUVECs cells with various concentrations of aqueous extract of saffron (50, 100, 200, and 400 µg/ml) 24 hr before the induction of oxidative stress produced a notable rise in miR-21 and miR-142-3p. The findings showed evidence of the aqueous extract of saffron’s capability to counteract oxidative stress through the miRNAs pathway (39).

A clinical trial study looked into the effects of taking a 50 mg saffron capsule twice daily for 6 weeks in patients with atherosclerosis. The findings revealed that saffron had a significant effect in decreasing miR-21 expression levels in patients with atherosclerosis in comparison to the placebo group (40). Research has established that miR-21 induces a decrease in the expression of peroxisome proliferator-activated receptor alpha (PPAR-α) and an augmented hazard of atherosclerosis. Saffron likely has a protective effect against atherosclerosis by decreasing the production of miR-21 and increasing the expression and functioning of PPAR-α (40).

*Effects of crocin*

The impact of miRNA-188 in cardiac remodeling has been uncovered. MiR-188 is primarily located in dendritic cells and is essential for cell differentiation. Evidence suggests that miR-188 can help reduce the severity of myocardial infarction by inhibiting the autophagic process through the ATG7 protein (67). Research has concluded that a decrease in miRNA-188 expression leads to autophagy in the heart during ischemic-reperfusion (67). A rat model of hyperhomocysteinemia (HHcy) caused by L-methionine has been used to investigate the effect of crocin (50 mg/kg body weight/day, IP) on miR-188 expression after HHcy induction for 4 weeks. The findings revealed that HHcy had a remarkable effect on lowering the production of miR-188, but crocin intervention was effective in avoiding the decrease. Based on the study, crocin was found to inhibit heart damage induced by HHcy in animals through the enhancement of miR-188 (41). 

MiR-126 is one of the miRNAs thought to have a pro-angiogenic impact, which is present in high quantities in endothelial cells (68). In several studies, it has been observed that miRNA-126 is reduced in patients with coronary artery disease (CAD), acute myocardial infarction (AMI), and heart failure (68, 69). On the other hand, miRNA-210 is also understood to be a miRNA that is associated with apoptosis and angiogenesis and primarily responds to hypoxic conditions (70). MiRNA-210 has a major impact on the formation of collateral circulation in AMI, leading to better clinical outcomes (71). The eight-week oral ingestion of crocin (50 mg/kg) has been considered in terms of its effects on the expression of miR-126 and miR-210 in rat heart tissue. The results of crocin intervention for eight weeks showed augmented miR-126 and miR-210 expression. Examination of the results indicated that pre-administration of crocin altered heart angiogenesis by raising Akt and ERK1/2 levels regulated by miR-126 and miR-210 (42). 

MiRNA-34a has been identified as a critical factor in various cardiac activities such as aging, apoptosis, fibrosis, autophagy, and inflammation and could be used as a therapeutic goal (72). Altered miRNA-34a concentrations have been noticed in different heart conditions such as myocardial infarction, heart failure, cardiovascular fibrotic remodeling, atherosclerosis, cardiomyopathy, atrial fibrillation, and hypertension (72). Elevated levels of miRNA-34a have been linked to the stimulation of apoptosis in MI cases by inhibiting silent information regulator 1 (SIRT1) (73). The protective effect of crocin on ischemia-reperfusion-induced left ventricular dysfunction and infarction has been explored in ischemia-reperfusion-induced primary cardiomyocytes and mouse models. Data indicated that by administering 10 μM crocin for 24 hr before hypoxia, the I/R-induced cardiomyocyte apoptosis was decreased, due to the diminishment of miR-34a expression. It appears that crocin’s protection from I/R-induced left ventricular dysfunction and infarct is due to decreased miR-34a and increased Sirt1, Nrf2, and heme oxygenase-1 (HO-1) expression (43).

In summary, saffron and Crocin’s effects on CVD have been demonstrated in experimental and human studies, including antioxidants, anti-apoptosis, and anti-atherosclerosis. It is interesting to note that the effects of saffron and crocin have been achieved through the modulation of different miRNAs such as miR-21, miR-142-3p, miR-188, miR-126, miR-210, and miR-34a (Table 1).

***Cancer disorders***

*Effects of crocin*

MiRNAs have been demonstrated to have a major part in numerous cellular functions associated with cancer, such as cancer induction, apoptosis, expansion of cancer, and cancer metastasis (74). miRNAs have been proven to be involved in the regulation of gene expression in both internal and external apoptotic pathways (75). It has been revealed that miR-15a is capable of activating the caspase 3/7 pathway in mouse cells and decreasing the survival rate of cancer cells. The target genes for miRNA-15a function may be Bcl-x_L_, naip5, fgfr2, and mybl2 (76). Evidence suggests that hsa-miR-15a has a pro-apoptotic effect by inhibiting growth and activating cell death (76). The effect of crocin in CO 88 BV 59-1 (type-B lymphomas infected by Epstein Barr) has been studied on the viability of the cells as well as on the expressions of miR-15a3 and miR-15a5 of the cells. Cells exposed to crocin concentrations ranging from 0.2 to 200 μM for three days showed a marked decrease in proliferation and a proportional increase in apoptosis. There was no noteworthy difference in miR-15a3p and miR-15a5p expression between the cells exposed to crocin and the control (45). 

The miR-320a has received attention for its potential involvement in cancer. Levels of miR-320a have been observed to be lower in non-small cell lung cancer (NSCLC), colorectal, osteosarcoma, and breast cancer (77). The findings have demonstrated that miR-320a has a cancer-inhibiting function, and its decreased concentrations have caused the development of malignancy (77). There was a negative relationship between miR-320a levels and cancer stages in colorectal cancer (78). Clinical indicators such as tumor types and lymph node metastases in NSCLC patients have been linked to lower concentrations of miR-320a (77). Studies of *in vitro* and *in vivo* experiments have indicated that crocin can produce anti-proliferative and apoptotic effects on cutaneous squamous cell carcinoma, as evidenced by the expression of miR-320a. An intervention with crocin (0, 1, 2, and 4 mM) was carried out for 24 to 48 hr on A431 and SCL-1 cell lines, cSCC human specimens, and BALB nude mice. An increased expression of miR-320a was observed in cSCC clinical samples. The utilization of crocin was verified to effectively suppress the growth of cSCC cells while inducing apoptosis. Crocin’s antitumor properties resulted from preventing the augmented production of miR-320a and stimulating ATG2B’s expression (46). Furthermore, crocin has been observed to modify the expression of Krüppel-like factor 5 (KLF5), hypoxia-inducible factor-1α (HIF-1α), and miR-320 in AGS and HGC-27 gastric cancer cells. Utilization of crocin (2 or 3 μM for a day) was associated with a reduction in KLF5 and HIF-1α expression and an increase in miR-320 expression. It was found that crocin exerted inhibitory effects on migration, invasion, and epithelial-mesenchymal transition (EMT) of gastric cancer cells by reducing the expression of KLF5, which was the target gene for miR-320 (50).

Studies have shown MiR-365 to have two distinct influences on cancer, with a larger presence in gastric cancer, cutaneous squamous cell carcinoma, and pancreatic cancer. On the other hand, in lung cancer, melanoma, colon cancer, osteosarcoma, and glioma, its levels are decreased (79). MiRNA expression has been utilized to explore the antitumor effects of crocin on the sensitive human cervical cancer cell line (OV2008). It was shown that crocin (0–4 mg/ml for 72 hr) together with citalopram had antiproliferation and anti-apoptotic effects in the cell line. The antitumor effects of crocin were likely due to the diminished miR-365 expression and the marked increase in the Bax/Bcl-2 ratio and p53 (47, 48). 

MiR-181 a, b, c, and d have demonstrated their ability to function as tumor suppressors or promoters of malignancy in multiple cancer types (80). In cancers such as hepatocellular carcinoma, pancreatic carcinoma, oral cancer, colorectal carcinoma, ovarian cancers, prostate cancer, breast cancer, neuroblastoma, and multiple myeloma, greater expression of miR-181 family has been observed, while in malignancies including pancreatic carcinoma, colorectal carcinoma, NSCLC, astrocytoma, glioma and glioblastoma, chronic myeloid leukemia (CML), and acute myeloid leukemia (AML), a decline in miR-181 family is apparent (80). miRNAs have revealed the anti-tumor effects of crocin on cervical cancer cells. Crocin (5 mg/ml for 48 hr) was used to treat the resistance of HELA/Ara-C cells, resulting in anti-proliferative effects and apoptosis induction. The results showed that crocin caused an anti-tumor effect by up-regulating the expression of miR-183a, whereas it did not affect the expression of miR-183b or miR-183c (49).

Although the majority of studies have demonstrated a tumor suppressor role for miR-34a, some have proposed its role as an oncomir. It has been uncovered that miR-34a is expressed at a lower level in certain cancers such as head and neck squamous cell carcinoma, and nasopharyngeal carcinomas (81). In contrast, higher expression of miR-34a has been observed in certain cancers such as oropharyngeal carcinoma and thyroid neoplasms (81). The anti-tumorigenic properties of crocin in papillary thyroid cancer (PTC) cells have been evaluated. Evidence has been provided that crocin has anti-proliferative and apoptosis-inducing impacts on PTC cells. Crocin significantly suppressed the expression of miR-34a-5p in PTC cells. The process of crocin causing anticancer effects was established through the decrease in miR-34a-5p and the increase in protein tyrosine phosphatase nonreceptor type 4 (PTPN4) (51). 

In summary, studies *in vitro* and *in vivo* have revealed that crocin positively affects cancer. Cancer has been associated with changes in miRNA expression. Remarkably, the effects of crocin are mainly achieved through the alteration of diverse miRNAs like miR-320a, miR-365, miR-181a, and miR-34a (Table 2).

***Diabetes disorders***

*The effects of crocin*

Studies have demonstrated a correlation between an elevated miR-210 in patients with diabetes mellitus (DM) and coronary artery disease. An imbalance of miR-210 impairs the angiogenesis process in diabetic individuals, leading to the exacerbation of cardiac and circulatory disorders through endothelial dysfunction (82). On the other hand, miR-126 is a significant factor in angiogenesis and preserving vascular integrity. MiR-126 causes angiogenesis effects by releasing angiogenesis factors such as hypoxia-inducible factor 1-alpha (HIF-1a), matrix metalloproteinases (MMPs), and vascular endothelial growth factor (VEGF) (83). An inhibition of VEGF caused by miRNA-126 has been observed to lessen diabetic retinopathy in mice (83). In an animal study, the effect of oral administration of crocin (50 mg/kg) for eight weeks on the expression of miR-210 and miR-126 in the heart tissue of type 2 diabetic rats induced by streptozotocin injection has been investigated. The results showed that the expression of miR-210 and miR-126 was significantly decreased in the heart tissue of diabetic rats. The intervention of crocin resulted in a marked rise in miR-210 and miR-126 in cardiac tissue, with the combination of the intervention and exercise showing marked effects on this rise (52). 

Studies have demonstrated that diabetic patients in the early stages of development are characterized by elevated levels of miR-204 as their beta cells are beginning to deteriorate. Inflammation of pancreatic beta cells has been linked to cell death by apoptosis and is accompanied by an elevation of miR-204 (84). It has come to light that miR-204 is unique to type 1 diabetes mellitus (T1DM), and no unusual values have been seen in type 2 diabetes mellitus (T2DM) (85). On the other hand, findings have revealed that miR-192 might be involved in the development of diabetes-related kidney issues, including diabetic nephropathy (86). MiR-29 is highly present in the islets of Langerhans and is a major factor in insulin secretion and beta-cell apoptosis (85). It is known that glucose stimulation in pre-diabetics leads to a rise in MiR-29 in beta cells, and this has a repressive effect on glucose-activated insulin secretion. Furthermore, raised amounts of miR-29 have been identified as a consequence of exposure to inflammatory cytokines (85). miR-216 is one of the miRNAs examined for its role in diabetes. It was discovered that miR-216 was overexpressed in prediabetic mice (87). In T1D, higher amounts of miR-216 have been documented. Preventing miR-216 has been noted to result in more beta-cell growth and higher levels of insulin secretion (87). Research has been conducted to determine the effect of crocin therapy on the expression of miRNAs 204, 216b, 192, and 29a in the pancreas tissue of T2DM rats caused by methylglyoxal. Results indicated that miRNAs 204, 216b, 192, and 29a were expressed to a greater degree in the pancreas tissue of diabetic rats. Taking crocin (15, 30, and 60 mg/kg) orally over four weeks blocked the increase of these miRNAs in the pancreatic tissue of diabetic rats (53). 

A different experiment looked into the effects of taking 15, 30, and 60 mg/kg/d of crocin orally over four weeks on the expression of miR-204, miR-192, and miR-29a in the kidneys of T2DM rats induced with methylglyoxal. A rise in the expression of miR-204 and miR-192 was noted in the kidney tissue of diabetic rats, whereas the expression of miR-29a decreased. Intervention with crocin notably decreased the expression of miR-204 and miR-192 and changed the expression of miR-29a (54). 

*Effects of crocetin*

Studies have demonstrated that the amount of miR-125b expressed in peripheral blood mononuclear cells (PBMCs) taken from diabetics is greater. It has been established that miR-125b prevents the procedure of apoptosis by controlling p53 activity and is important in cell proliferation (88, 89). Also, miR-125b has led to increased insulin sensitivity and increased beta cell function (88, 89). In an *in vivo* study using mice with diabetic retinopathy, the influence of crocetin (100 mg/kg administered three times over four days) was examined, as well as the effect of crocetin (50 μM, 100 μM, and 200 μM used for two days) on human retinal microvascular endothelial cells (hRMECs) in an *in vitro* study, with a focus on the expression of miR-125b-5p. The data revealed that miR-125b-5p was significantly diminished when the glucose concentration was high, but crocetin treatment was able to effectively prevent the decrease in miR-125b-5p expression (55).

In summary, the positive effects of crocin and crocetin were observed in *in vitro* studies of diabetic conditions. The effects of crocin and crocetin are caused by the modulation of different miRNAs such as miR-210, miR-126, miR-204, miR-216b, miR-192, miR-29a, and miR-125b (Table 3).

***Liver disorders***

*The effects of crocin*

Liver tissue exhibits a significantly elevated expression of miR-122. MiR-122 has been demonstrated to play an essential role in the development, differentiation, homeostasis, and functions of the liver (90). Interestingly, the role of miR-122 in the metabolism of fatty acids and cholesterol has also been revealed (91). MiR-122 has been considered one of the therapeutic targets in various liver diseases such as fibrosis, viral hepatitis, steatosis, and *hepatocellular carcinoma *(HCC). Decreased expression of miR-122 has been reported in liver fibrosis, liver inflammation, non-alcoholic steatohepatitis, and liver cancer (90). Additionally, higher levels of miR-34a have been observed in a variety of liver diseases including HCC, hepatitis C virus (HCV) infection, hepatic fibrosis, alcoholic liver disease, and nonalcoholic fatty liver disease (NAFLD) (92). Inhibiting miR-34a has resulted in a decrease in triglyceride (TG), liver index, liver steatosis, and plasma aspartate aminotransferase (AST) levels, which confirms the therapeutic importance of miR-34a in liver diseases (92). The protective effects of pretreatment with crocin (200 mg/Kg) for seven consecutive days in liver ischemia-reperfusion (I/R) injury in rats have been investigated through miRNAs. The study demonstrated that miR-122 and miR-34a expression were elevated in animals with liver I/R damage. Crocin treatment prevented the increase in miR-122 and miR-34a expression, whereas it enhanced the expression of nuclear factor erythroid 2–related factor 2 (Nrf2) in liver tissue (58).

A further study was conducted to investigate the protective effect of crocin in hepatic I/R injury in rats by assessing the change in miR-122 and miR-34a expression. It was observed that miR-122 and miR-34a expression were higher in rats with I/R-induced hepatic injury. Seven days of 200 mg/kg intraperitoneal crocin administration decreased the levels of miR-122, miR-34a, and p53 and prevented the liver from damage (61). 

The protective effects of crocin have also been investigated in Bisphenol A (BPA)-induced liver toxicity in rats through miR-122 expression. Treatment with crocin (5, 10, or 20 mg/kg) was done intraperitoneally for thirty days. Liver damage, increased activity of oxidants, and up-regulation of miR-122 were observed in BPA-treated rats. Treatment with crocin, especially 20 mg/kg, prevented BPA-induced liver damage and led to the down-regulation of miR-122 (59). 

MiR-29 and MiR-9 are two miRNAs that are expressed in considerable quantities in the liver and are also known as antifibrotic miRNAs (93, 94). In addition, miR-29 has been suggested to control pathways connected to biliary atresia. (93). A reduction in miR-29 and miR-9 has been noticed in diseases like liver fibrosis/cirrhosis and HCC. (93, 94). To assess the protective effect of crocin on rats with cisplatin-induced hepatotoxicity, miR-29 and miR-9 were studied. It was discovered that cisplatin-induced hepatotoxicity led to a significant decrease in the expression of miR-29 and miR-9. Administering crocin (200 mg/kg IP) for a week significantly prevented the reduction in miR-29 and miR-9 expression (60). 

In summary, in studies that have investigated liver damage *in vivo*, it has been determined that crocin had damage prevention or recovery effects. The beneficial effects of crocin are mediated through the modulation of various miRNAs such as miR-122, miR-34a, miR-9, and miR-29 (Table 4). 

***Central and peripheral nervous system disorders***

*Effects of saffron*

The role of miRNAs-133, 9, 29b-1, 29a, and 133b has been investigated in various psychological disorders. Results have indicated a reduction in miRNAs-133, 9, 29b-1, 29a, and 133b accompanied by an elevated level of ceramides and amyloid-beta protein (95, 96). On the other hand, miRNA expression has been disrupted in illnesses such as Parkinson’s, Huntington, multiple sclerosis, and Alzheimer’s (95). Elevated levels of miRNA-133 correlated with severe depression and diabetes in mice (97). In rats with trimethyltin (TMT)-induced Alzheimer’s disease (AD), the therapeutic effects of aqueous extract of saffron (25 mg/kg daily, IP) for eight weeks on the expression of miR-133bFC and miR-29aFC in hippocampus tissue have been investigated. Saffron therapy was associated with a reduced expression of miR-133bFC in comparison to rats with AD, however, it had no effect on the expression of miR-29aFC (56). 

*Effects of crocin*

Data from multiple studies have demonstrated the importance of miR-7 in the functioning of the pituitary gland, optic nervous system, cerebral cortex, and other brain regions (98). An increase in the expression of miR-7 has been discovered to be associated with brain disorders like Alzheimer’s, schizophrenia, Lewy body dementia (DLB), and cerebral artery malformation (98). On the other hand, miR-221’s activity has been explored in many biological processes. It has been observed that miR-221 is increased in a variety of cancers such as HCC, prostate adenocarcinoma, and colorectal carcinoma (99). Further, it has been observed that lower levels of miR-221 are linked to psychological disorders like Parkinson’s disease (99). Researchers have studied how crocin can provide a protective effect against ROT-induced Parkinson’s in rats by regulating miR-7 and miR-221 expression. In rodent models with ROT-induced Parkinson’s disease (PD), miR-7 and miR-221 expression were seen to be lower in the striatum and substantia nigra pars compacta. Administering 30 mg/kg/day of crocin intraperitoneally for 30 days caused an increase in the expression of miR-7 and miR-221 (57). 

Table 5 demonstrates the effect of saffron and its constituents on miRNA levels in nerve system disorders and other disorders and a potential mechanism.

***Other disorders***

*Effects of crocin*

An animal study has been conducted to analyze the impact of crocin on miR-146a and miR-106a expression in the lung tissue of ovalbumin (OVA)-sensitized mice. The lung tissue of asthmatic model mice showed increased expression of miR-146a and miR-106a. Administering crocin (25, 50, and 100 mg/kg) for five days was found to prevent the rise in the expression of miR-146a and miR-106a. Notably, the highest concentrations of crocin (50 and 100 mg-kg) yielded the most pronounced results (37).

A clinical trial examined the influence of a 4-month treatment of crocin (15 mg daily) on osteoarthritis patients concerning the expression of miR-146a, miR-155, miR-223, and miR-21 in PBMC samples. Analysis indicated that after the application of crocin, the level of miR-21 was considerably reduced, while the expression of miR-155 was enhanced. There were no noticeable changes in the amounts of miRNA-146a and miRNA-223 in patients with osteoarthritis (62).

*Effects of crocetin*

It has been studied what effect crocetin has on miR-21, miR-127, and miR-132 in the context of kidney damage resulting from ischemia-reperfusion (I/R). It was noted that miR-21 and miR-127 were more expressed in the kidney tissue after it was subjected to I/R-induction, and intervention with crocetin (50 mg/kg for 24 hr, IP) prevented the rise of miRNAs (44).

Table 5 demonstrates the effect of saffron and its constituents on miRNA levels in nerve system disorders and other disorders and a potential mechanism.

**Figure 2:**
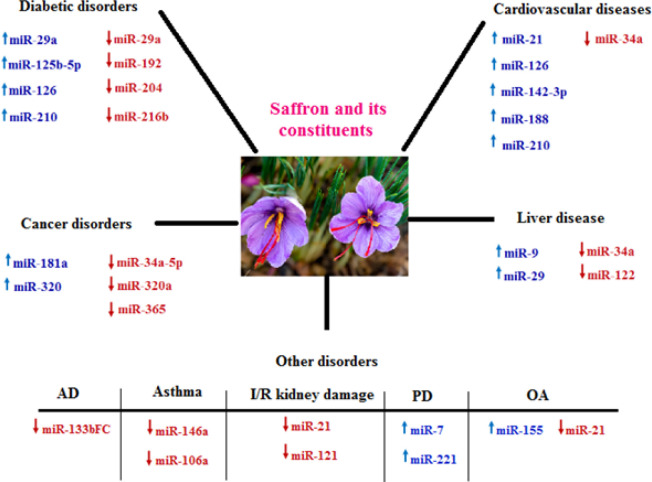
A summary of the effects of saffron and its active ingredients in various disorders

AD: Alzheimer's disease; I/R: ischemia/reperfusion; PD: Parkinson's disease; OA: osteoarthritis.

Figure 2 shows a summary of the effects of saffron and its active ingredients on various disorders. 

**Table 1:** A summary of the effects of saffron and its active ingredients on miRNAs in cardiovascular diseases

**Preparations**	**Study design**	**Dose**	**Effects**	**References**
AE of saffron	TBHP and AAPH induced oxidative stress on HUVECs	50, 100, 200, and 400 µg/ml for 24 hr	↑ miRNA-21 ↑ miRNA142-3p	(39)
saffron	AS patients	50 mg twice a day for 6 weeks, orally	↑ mR-21	(40)
Crocin	LM-induced HHcy in rats	50 mg/kg/day, for 4 weeks, IP	↑ miR-188	(41)
Crocin	Heart tissue of Wistar rat	50 mg/kg for 8 weeks, orally	↑ miR-126 ↑ miR-210	(42)
Crocin	I/R-induced cardiomyocyte apoptosis	10 μM for 24 hr before hypoxia	↓ miR-34a	(43)

AAPH: 2,2-azobis (2-amidinopropane) dihydrochloride, AE: aqueous extract, AS: atherosclerosis, HUVECs: human vascular endothelial cells, HHcy: hyperhomocysteinemia, IP: intraperitoneal, I/R: ischemia-reperfusion, LM: L-methionine, TBHP: tertbutyl hydroperoxide.

**Table 2:** A summary of the effects of saffron and its active ingredients on miRNAs in cancer disorders

**Preparations**	**Study design**	**Dose**	**Effects**	**References**
Crocin	type B lymphoma infected with Epstein-Barr virus	0.2 to 200 μM for 72 hr	No effect on miR-15a5p No effect on miR-15a3p	(45)
Crocin	A431 and SCL-1 cell lines	0, 1, 2, and 4 mM for 48 hr	↓ miR-320a	(46)
Crocin	AGS and HGC-27 cell lines	2 or 3 μM for 24 hr	↑ miR-320	(50)
Crocin	OV2008 cell lines	0-4 mg/ml for 72 hr	↓ miR-365	(48)
Crocin and CDDP	OV2008 cell line	Crocin: 0-4 mg/ml CDDP: 0.003 mg/ml for 72 hr	↓ miR-365	(47)
Crocin	HELA/Ara-C cell lines	5 mg/ml for 48 hr	↑ miR-181a No effect on miR-181b No effect on miR-181c	(49)
Crocin	PTC cells	-	↓ miR-34a-5p	(51)

A431: epidermoid carcinoma cell line, AGS: a human gastric adenocarcinoma cell line, HGC-27: human gastric carcinoma, OV2008: human cervical cancer cell line, PTC: papillary thyroid cancer, SCL-1: squamous cell carcinoma cell line 1

**Table 3:** A summary of the effects of saffron and its active ingredients on miRNAs in diabetic disorders

**Preparations**	**Study design**	**Dose**	**Effects**	**References**
Crocin	STZ-induced diabetic rats	50 mg/kg for 8 weeks, orally	↑ miR-210 ↑ miR-126	(52)
Crocin	MG-induced diabetic rats	15, 30, and 60 mg/kg for 4 weeks, orally	↓ miR-204 ↓ miR-216b ↓ miR-192 ↓ miR-29a	(53)
Crocin	MG-induced diabetic rats	15, 30, and 60 mg/kg/d for 4 weeks, orally	↓ miR-204 ↓ miR-192 ↑ miR-29a	(54)
Crocetin	Hg-induced DR on hRMECs and 293T cells	50 μM, 100 μM and 200 μM for 48 h	↑ miR-125b-5p	(55)
Crocetin	STZ-induced DR in mice	100 mg/kg for 4 days three times a day, orally	↑ miR-125b-5p	(55)

DR: diabetic retinopathy, Hg: mercury, hRMECs: human retinal microvascular endothelial cells, IP: intraperitoneal, MG: methylglyoxal, STZ: streptozotocin

**Table 4:** A summary of the effects of saffron and its active ingredients on miRNAs in hepatic disorders

**Preparations**	**Study design**	**Dose**	**Effects**	**References**
Crocin	I/R-induced hepatic injury rats	200 mg/kg for 7 days, IP	↓ miR-122 ↓ miR-34a	(58)
Crocin	I/R-induced hepatic injury rats	200 mg/kg for 7 days, IP	↓ miR-122 ↓ miR-34a	(61)
Crocin	BPA-induced liver toxicity in rats	5, 10, or 20 mg/kg for thirty days, IP	↓ miR-122	(59)
Crocin	CP-induced hepatotoxicity in rats	200 mg/kg for 7 days, IP	↑ miRNA-9 ↑ miR-29	(60)

BPA: bisphenol A, CP: cisplatin, IP: intraperitoneal, I/R: ischemia-reperfusion

**Table 5:** A summary of the effects of saffron and its active ingredients on miRNAs in Alzheimer's and Parkinson's diseases and other disorders

**Preparations**	**Study design**	**Dose**	**Effects**	**References**
AE of saffron	TMT-induced AD in rat	25 mg/kg daily for 8 weeks, IP	↓ miR-133bFC No effect on miR-29aFC	(56)
Crocin	ROT-induced PD in rats	30 mg/kg/day for 30 days, IP	↑ miR-7 ↑ miR-221	(57)
Crocin	OVA-sensitized mice	25, 50, and 100 mg/kg for 5 days, IP	↓ miR-146a ↓ miR-106a	(37)
Crocin	OA patients	15 mg per day for 4 months, orally	↓ miR-21 ↑ miR-155 No effect on miR-146a No effect on miR-223	(62)
Crocetin	I/R-induced kidney damage in rat	50 mg/kg/day for 24 hr, IP	↓ miR-21 ↓ miR-127 No effect on miR-132	(44)

AD: alzheimer's disease, AE: aqueous extract, IP: intraperitoneal, I/R: ischemia/reperfusion, OA: osteoarthritis, OVA: ovalbumin, PD: parkinson's disease, ROT: rotenone, TMT: trimethyltin

## Conclusion

miRNA dysregulation has become a viable therapeutic option for various diseases. Alterations in miRNA levels, either up- or down-regulated, can influence the magnitude of diseases. Studies have indicated that saffron and its active ingredients can modify miRNA levels in several disorders, including cardiovascular, diabetic, neurological, and respiratory disorders, and cancers. An active ingredient of saffron, safranal, has been studied in numerous studies but none have focused on miRNAs. The role of safranal regarding miRNAs should be considered more in future studies. This review showed saffron’s ability to modify miRNA expression, but further studies are necessary for a more complete analysis. 

## Authors’ Contributions

MR A and H G contributed to conceptualization, methodology, data analysis, manuscript preparation, and revising the manuscript. A A, F A, and E K helped extract data, prepare the original draft, review, and edit.

## Declaration of Competing Interest

The authors declare that they have no competing interests.

## Ethics Approval

This study was conducted after approval by the ethics committee of the Ardabil University of Medical Sciences, Iran (IR.ARUMS.REC.1401.242).

## Funding

There was no funding for the study.

## Consent for Publication

All authors consent to publication of this article in this journal.

## Data Availability

Not applicable.

## References

[1] O’Brien J, Hayder H, Zayed Y, Peng C (2018). Overview of microRNA biogenesis, mechanisms of actions, and circulation. Front Endocrinol (Lausanne).

[2] Akhavanakbari G, Babapour B, Alipour MR, Keyhanmanesh R, Ahmadi M, Aslani MR (2019). Effect of high fat diet on NF-kB microRNA146a negative feedback loop in ovalbumin-sensitized rats. Biofactors.

[3] Vasudevan S (2012). Posttranscriptional upregulation by microRNAs. Wiley Interdiscip Rev RNA.

[4] Dharap A, Pokrzywa C, Murali S, Pandi G, Vemuganti R (2013). MicroRNA miR-324-3p induces promoter-mediated expression of RelA gene. PLoS One.

[5] Bottini S, Hamouda-Tekaya N, Mategot R, Zaragosi L-E, Audebert S, Pisano S (2017). Post-transcriptional gene silencing mediated by microRNAs is controlled by nucleoplasmic Sfpq. Nat Commun.

[6] Denli AM, Tops BB, Plasterk RH, Ketting RF, Hannon GJ (2004). Processing of primary microRNAs by the microprocessor complex. Nature.

[7] Ruby JG, Jan CH, Bartel DP (2007). Intronic microRNA precursors that bypass Drosha processing. Nature.

[8] Barrey E, Saint-Auret G, Bonnamy B, Damas D, Boyer O, Gidrol X (2011). Pre-microRNA and mature microRNA in human mitochondria. PLoS One.

[9] Chen X, Ba Y, Ma L, Cai X, Yin Y, Wang K (2008). Characterization of microRNAs in serum: A novel class of biomarkers for diagnosis of cancer and other diseases. Cell Res.

[10] Sohn W, Kim J, Kang SH, Yang SR, Cho J-Y, Cho HC (2015). Serum exosomal microRNAs as novel biomarkers for hepatocellular carcinoma. Exp Mol Med.

[11] Gallo A, Tandon M, Alevizos I, Illei GG (2012). The majority of microRNAs detectable in serum and saliva is concentrated in exosomes. PLoS One.

[12] Turchinovich A, Weiz L, Burwinkel B (2012). Extracellular miRNAs: the mystery of their origin and function. Trends Biochem Sci.

[13] Hammond SM (2015). An overview of microRNAs. Adv Drug Deliv Rev.

[14] Zhou S-s, Jin J-p, Wang J-q, Zhang Z-g, Freedman JH, Zheng Y (2018). miRNAS in cardiovascular diseases: Potential biomarkers, therapeutic targets and challenges. Acta Pharmacol Sin.

[15] Barbarotto E, Schmittgen TD, Calin GA (2008). MicroRNAs and cancer: profile, profile, profile. Int J Cancer.

[16] Xiao L, Wang J-Y (2014). RNA-binding proteins and microRNAs in gastrointestinal epithelial homeostasis and diseases. Curr Opin Pharmacol.

[17] Hussein M, Magdy R (2021). MicroRNAs in central nervous system disorders: Current advances in pathogenesis and treatment. Egypt J Neurol Psychiatr Neurosurg.

[18] Deiuliis J (2016). MicroRNAs as regulators of metabolic disease: pathophysiologic significance and emerging role as biomarkers and therapeutics. Int J Obes.

[19] Javandoost A, Afshari A, Nikbakht-Jam I, Khademi M, Eslami S, Nosrati M (2017). Effect of crocin, a carotenoid from saffron, on plasma cholesteryl ester transfer protein and lipid profile in subjects with metabolic syndrome: A double blind randomized clinical trial. ARYA Atheroscler.

[20] Kothari D, Thakur R, Kumar R (2021). Saffron (Crocus sativus L ): Gold of the spices—a comprehensive review. Hortic Environ Biotechnol.

[21] El Midaoui A, Ghzaiel I, Vervandier-Fasseur D, Ksila M, Zarrouk A, Nury T (2022). Saffron (Crocus sativus L ): A source of nutrients for health and for the treatment of neuropsychiatric and age-related diseases. Nutrients.

[22] Ghaffari S, Roshanravan N (2019). Saffron; An updated review on biological properties with special focus on cardiovascular effects. Biomed Pharmacother.

[23] Kyriakoudi A, Tsimidou MZ, O’Callaghan YC, Galvin K, O’Brien NM (2013). Changes in total and individual crocetin esters upon in vitro gastrointestinal digestion of saffron aqueous extracts. J Agric Food Chem.

[24] Lautenschläger M, Sendker J, Hüwel S, Galla H, Brandt S, Düfer M (2015). Intestinal formation of trans-crocetin from saffron extract (Crocus sativus L ) and in vitro permeation through intestinal and blood brain barrier. Phytomedicine.

[25] Boskabadi M, Aslani M, Mansouri F, Ameri S (2007). Relaxant effect of Satureja hortensis on guinea pig tracheal chains and its possible mechanism (s). Daru J Pharm Sci.

[26] Saadat S, Aslani MR, Ghorani V, Keyhanmanesh R, Boskabady MH (2021). The effects of Nigella sativa on respiratory, allergic and immunologic disorders, evidence from experimental and clinical studies, a comprehensive and updated review. Phytother Res.

[27] Khazdair MR, Saadat S, Aslani MR, Shakeri F, Boskabady MH (2021). Experimental and clinical studies on the effects of Portulaca oleracea L and its constituents on respiratory, allergic, and immunologic disorders, a review. Phytother Res.

[28] Ghasemi Z, Rezaee R, Aslani MR, Boskabady MH (2021). Anti-inflammatory, anti-oxidant, and immunomodulatory activities of the genus Ferula and their constituents: A review. Iran J Basic Med Sic.

[29] Abedi A, Ghobadi H, Sharghi A, Iranpour S, Fazlzadeh M, Aslani MR (2023). Effect of saffron supplementation on oxidative stress markers (MDA, TAC, TOS, GPx, SOD, and pro-oxidant/antioxidant balance): An updated systematic review and meta-analysis of randomized placebo-controlled trials. Front Med.

[30] Razavi BM, Hosseinzadeh H, Abnous K, Imenshahidi M (2014). Protective effect of crocin on diazinon induced vascular toxicity in subchronic exposure in rat aorta ex-vivo. Drug Chem Toxicol.

[31] Rahimi G, Shams S, Aslani MR (2022). Effects of crocin supplementation on inflammatory markers, lipid profiles, insulin and cardioprotective indices in women with PCOS: A randomized, double-blind, placebo-controlled trial. Phytother Res.

[32] Karuppusamy A, Krishnan A, Subramanian P, Anathy V (2022). Oxidative stress related cellular metabolism in lung health and diseases. Front Pharmacol.

[33] Ghobadi H, Abdollahi N, Madani H, Aslani MR (2022). Effect of crocin from saffron (Crocus sativus L ) supplementation on oxidant/antioxidant markers, exercise capacity, and pulmonary function tests in COPD patients: A randomized, double-blind, placebo-controlled trial. Front Pharmacol.

[34] Aslani MR, Amani M, Masrori N, Boskabady MH, Ebrahimi HA, Chodari L (2022). Crocin attenuates inflammation of lung tissue in ovalbumin-sensitized mice by altering the expression of endoplasmic reticulum stress markers. Biofactors.

[35] Boskabady M, Aslani M (2006). Relaxant effect of Crocus sativus (saffron) on guinea-pig tracheal chains and its possible mechanisms. J Pharm Pharmacol.

[36] Saadat S, Yasavoli M, Gholamnezhad Z, Aslani MR, Boskabady MH (2019). The relaxant effect of crocin on rat tracheal smooth muscle and its possible mechanisms. Iran J Pharm Res.

[37] Aslani MR, Jafari Z, Rahbarghazi R, Rezaie J, Delkhosh A, Ahmadi M (2022). Effects of crocin on T-bet/GATA-3 ratio, and miR-146a and miR-106a expression levels in lung tissue of ovalbumin-sensitized mice. Iran J Basic Med Sic.

[38] Aslani MR, Abdollahi N, Matin S, Zakeri A, Ghobadi H (2023). Effect of crocin of Crocus sativus L on serum inflammatory markers (IL-6 and TNF-α) in COPD patients: A randomized, double-blind, placebo-controlled trial. Br J Nutr.

[39] Rodriguez-Ruiz V, Barzegari A, Zuluaga M, Zunooni-Vahed S, Rahbar-Saadat Y, Letourneur D (2016). Potential of aqueous extract of saffron (Crocus sativus L ) in blocking the oxidative stress by modulation of signal transduction in human vascular endothelial cells. J Funct Foods.

[40] Khatir SA, Bayatian A, Barzegari A, Roshanravan N, Safaiyan A, Pavon-Djavid G (2018). Saffron (Crocus sativus L ) supplements modulate circulating microRNA (miR-21) in atherosclerosis patients; a randomized, double-blind, placebo-controlled trial. Iran Red Crescent Med J.

[41] Hussein SA, Ahmed TE, Amin A, Ali AH (2019). The anti-inflammatory and anti-apoptotic role of crocin against expression of MMp-9, TIMP-1, MCP-1, caspase-3, PPARα in heart tissue, and metamorphoses of microRNA 188 in hyperhomocysteinemic rats. Recent Adv Biol Med.

[42] Ghorbanzadeh V, Mohammadi M, Dariushnejad H, Abhari A, Chodari L, Mohaddes G (2017). Cardioprotective effect of crocin combined with voluntary exercise in rat: Role of mir-126 and mir-210 in heart angiogenesis. Arq Bras Cardiol.

[43] Wang X, Yuan B, Cheng B, Liu Y, Zhang B, Wang X (2019). Crocin alleviates myocardial ischemia/reperfusion-induced endoplasmic reticulum stress via regulation of miR-34a/Sirt1/Nrf2 pathway. Shock.

[44] Michael CP, Derpapas M, Aravidou E, Sofopoulos M, Michael P, Polydorou A (2020). The carotenoid compound of saffron crocetin alleviates effects of ischemia reperfusion injury via a mechanism possibly involving MiR-127. Cureus.

[45] Sotoodeh Jahromi A, Moradzadeh M, Kargar M, Kafilzadeh F, Jamalidoust M (2020). Effect of crocin on miRNA-15a expression in EBV infected transformed B cell. Pars J Med Sci.

[46] Bi X, Jiang Z, Luan Z, Qiu D (2021). Crocin exerts anti-proliferative and apoptotic effects on cutaneous squamous cell carcinoma via miR-320a/ATG2B. Bioengineered.

[47] Mollaei H, Hoshyar R, Abedini MR, Safaralizadeh R (2019). Crocin enhances cisplatin-induced chemosensitivity in human cervical cancer cell line. Int J Cancer Manag.

[48] Mollaei H, Safaralizadeh R, Babaei E, Abedini MR, Hoshyar R (2017). The anti-proliferative and apoptotic effects of crocin on chemosensitive and chemoresistant cervical cancer cells. Biomed Pharmacother.

[49] Xu L, Song J-D (2022). Crocin reversed the antitumor effects through up-regulation of MicroRNA-181a in cervical cancer cells. Food Sci Technol.

[50] Zhou Y, Xu Q, Shang J, Lu L, Chen G (2019). Crocin inhibits the migration, invasion, and epithelial-mesenchymal transition of gastric cancer cells via miR-320/KLF5/HIF-1α signaling. J Cell Physiol.

[51] Tang Y, Yang H, Yu J, Li Z, Xu Q, Ding B (2022). Crocin induces ROS-mediated papillary thyroid cancer cell apoptosis by modulating the miR-34a-5p/PTPN4 axis in vitro. Toxicol Appl Pharmacol.

[52] Dariushnejad H, Chodari L, Ghorbanzadeh V (2020). The combination effect of voluntary exercise and crocin on angiogenic miRNAs in high-fat diet/low-dose STZ-induced type2 diabetes in Rats: miR-126 and miR-210. Pharm Sci.

[53] Radmehr V, Ahangarpour A, Mard SA, Khorsandi L (2022). Crocin ameliorates microRNAs-associated ER stress in type 2 diabetes induced by methylglyoxal. Iran J Basic Med Sic.

[54] Radmehr V, Ahangarpour A, Mard SA, Khorsandi L (2022). Crocin attenuates endoplasmic reticulum stress in methylglyoxal-induced diabetic nephropathy in male mice: MicroRNAs alterations and glyoxalase 1-Nrf2 signaling pathways. Iran J Basic Med Sic.

[55] Chen Q, Xi X, Ma J, Wang X, Xia Y, Wang X (2022). The mechanism by which crocetin regulates the lncRNA NEAT1/miR-125b-5p/SOX7 molecular axis to inhibit high glucose-induced diabetic retinopathy. Exp Eye Res.

[56] Negarandeh Z, Salamat KM, Hosseini SA (2021). The effect of eight weeks of endurance training with saffron on miR133bFC, miR29aFC in the hippocampus tissue and depression in rats with alzheimer’s disease. Jundishapur J Nat Pharm Prod.

[57] Salama RM, Abdel-Latif GA, Abbas SS, Hekmat M, Schaalan MF (2020). Neuroprotective effect of crocin against rotenone-induced Parkinson’s disease in rats: Interplay between PI3K/Akt/mTOR signaling pathway and enhanced expression of miRNA-7 and miRNA-221. Neuropharmacology.

[58] Akbari G, Mard SA, Dianat M, Mansouri E (2017). The hepatoprotective and microRNAs downregulatory effects of crocin following hepatic ischemia-reperfusion injury in rats. Oxid Med Cell Longev.

[59] Hassani FV, Mehri S, Abnous K, Birner-Gruenberger R, Hosseinzadeh H (2017). Protective effect of crocin on BPA-induced liver toxicity in rats through inhibition of oxidative stress and downregulation of MAPK and MAPKAP signaling pathway and miRNA-122 expression. Food Chem Toxicol.

[60] Khedr L, Rahmo RM, Farag DB, Schaalan MF, Hekmat M (2020). Crocin attenuates cisplatin-induced hepatotoxicity via TLR4/NF-κBp50 signaling and BAMBI modulation of TGF-β activity: Involvement of miRNA-9 and miRNA-29. Food Chem Toxicol.

[61] Mard SA, Akbari G, Dianat M, Mansouri E (2017). Protective effects of crocin and zinc sulfate on hepatic ischemia-reperfusion injury in rats: a comparative experimental model study. Biomed Pharmacother.

[62] Mohebbi M, Atabaki M, Tavakkol-Afshari J, Shariati-Sarabi Z, Poursamimi J, Mohajeri SA (2022). Significant effect of crocin on the gene expression of microRNA-21 and microRNA-155 in patients with osteoarthritis. Iran J Allergy Asthma Immunol.

[63] Xu X, Kriegel AJ, Jiao X, Liu H, Bai X, Olson J (2014). miR-21 in ischemia/reperfusion injury: A double-edged sword?. Physiol Genomics.

[64] Cheng Y, Zhang C (2010). MicroRNA-21 in cardiovascular disease. J Cardiovasc Transl Res.

[65] Kumar S, Kim CW, Simmons RD, Jo H (2014). Role of flow-sensitive microRNAs in endothelial dysfunction and atherosclerosis: mechanosensitive athero-miRs. Arterioscler Thromb Vasc Biol.

[66] Sharma S, Liu J, Wei J, Yuan H, Zhang T, Bishopric NH (2012). Repression of miR-142 by p300 and MAPK is required for survival signalling via gp130 during adaptive hypertrophy. EMBO Mol Med.

[67] Wang K, Liu C-Y, Zhou L-Y, Wang J-X, Wang M, Zhao B (2015). APF lncRNA regulates autophagy and myocardial infarction by targeting miR-188-3p. Nat Commun.

[68] Qu M-J, Pan J-J, Shi X-J, Zhang Z-J, Tang Y-H, Yang G-Y (2018). MicroRNA-126 is a prospective target for vascular. Neuroimmunol Neuroinflammation.

[69] Ali W, Mishra S, Rizvi A, Pradhan A, Perrone MA (2021). Circulating microRNA-126 as an independent risk predictor of coronary artery disease: a case-control study. EJIFCC.

[70] Dang K, Myers KA (2015). The role of hypoxia-induced miR-210 in cancer progression. Int J Mol Sci.

[71] Fan ZG, Qu XL, Chu P, Gao YL, Gao XF, Chen SL (2018). MicroRNA-210 promotes angiogenesis in acute myocardial infarction. Mol Med Report.

[72] Hua C-C, Liu X-M, Liang L-R, Wang L-F, Zhong J-C (2022). Targeting the microRNA-34a as a novel therapeutic strategy for cardiovascular diseases. Front Cardiovasc Med.

[73] Dong F, Dong S, Liang Y, Wang K, Qin Y, Zhao X (2019). MiR-34a promotes myocardial infarction in rats by inhibiting the activity of SIRT1. Eur Rev Med Pharmacol Sci.

[74] Liu J, Zheng M, Tang Yl, Liang Xh, Yang Q (2011). MicroRNAs, an active and versatile group in cancers. Int J Oral Sci.

[75] Garofalo M, Quintavalle C, Di Leva G, Zanca C, Romano G, Taccioli C (2008). MicroRNA signatures of TRAIL resistance in human non-small cell lung cancer. Oncogene.

[76] Druz A, Chen Y-C, Guha R, Betenbaugh M, Martin SE, Shiloach J (2013). Large-scale screening identifies a novel microRNA, miR-15a-3p, which induces apoptosis in human cancer cell lines. RNA Biol.

[77] Khandelwal A, Sharma U, Barwal TS, Seam RK, Gupta M, Rana MK (2021). Circulating miR-320a acts as a tumor suppressor and prognostic factor in non-small cell lung cancer. Front Oncol.

[78] Fang Z, Tang J, Bai Y, Lin H, You H, Jin H (2015). Plasma levels of microRNA-24, microRNA-320a, and microRNA-423-5p are potential biomarkers for colorectal carcinoma. J Exp Clin Cancer Res.

[79] Zhu Y, Zhao H, Rao M, Xu S (2017). MicroRNA-365 inhibits proliferation, migration and invasion of glioma by targeting PIK3R3. Oncol Rep.

[80] Indrieri A, Carrella S, Carotenuto P, Banfi S, Franco B (2020). The pervasive role of the miR-181 family in development, neurodegeneration, and cancer. Int J Mol Sci.

[81] Kalfert D, Ludvikova M, Pesta M, Ludvik J, Dostalova L, Kholová I (2020). Multifunctional roles of miR-34a in cancer: A review with the emphasis on head and neck squamous cell carcinoma and thyroid cancer with clinical implications. Diagnostics.

[82] Yin C, Lin X, Sun Y, Ji X (2020). Dysregulation of miR-210 is involved in the development of diabetic retinopathy and serves a regulatory role in retinal vascular endothelial cell proliferation. Eur J Med Res.

[83] Pishavar E, Behravan J (2017). miR-126 as a therapeutic agent for diabetes mellitus. Curr Pharm Des.

[84] Grieco FA, Schiavo AA, Brozzi F, Juan-Mateu J, Bugliani M, Marchetti P (2019). The microRNAs miR-211-5p and miR-204-5p modulate ER stress in human beta cells. J Mol Endocrinol.

[85] Angelescu MA, Andronic O, Dima SO, Popescu I, Meivar-Levy I, Ferber S (2022). miRNAs as biomarkers in diabetes: moving towards precision medicine. Int J Mol Sci.

[86] Baltaci OF, Çolakoğlu Ş, Amuran GG, Aydin N, Sargin M, Korkmaz AK (2018). Exploring the role of miRNAs in the diagnosis of MODY3. Turk J Med Sci.

[87] Wang P, Liu Q, Zhao H, Bishop JO, Zhou G, Olson LK (2020). miR-216a-targeting theranostic nanoparticles promote proliferation of insulin-secreting cells in type 1 diabetes animal model. Sci Rep.

[88] Yu C-Y, Yang C-Y, Rui Z-L (2019). MicroRNA-125b-5p improves pancreatic β-cell function through inhibiting JNK signaling pathway by targeting DACT1 in mice with type 2 diabetes mellitus. Life Sci.

[89] Shen Y, Xu H, Pan X, Wu W, Wang H, Yan L (2017). miR34a and miR125b are upregulated in peripheral blood mononuclear cells from patients with type 2 diabetes mellitus. Exp Ther Med.

[90] Bandiera S, Pfeffer S, Baumert TF, Zeisel MB (2015). miR-122–a key factor and therapeutic target in liver disease. J Hepatol.

[91] Esau C, Davis S, Murray SF, Yu XX, Pandey SK, Pear M (2006). miR-122 regulation of lipid metabolism revealed by in vivo antisense targeting. Cell Metab.

[92] Ding J, Li M, Wan X, Jin X, Chen S, Yu C (2015). Effect of miR-34a in regulating steatosis by targeting PPARα expression in nonalcoholic fatty liver disease. Sci Rep.

[93] Huang Y-H, Yang Y-L, Wang F-S (2018). The role of miR-29a in the regulation, function, and signaling of liver fibrosis. Int J Mol Sci.

[94] Sun J, Zhang H, Li L, Yu L, Fu L (2017). MicroRNA-9 limits hepatic fibrosis by suppressing the activation and proliferation of hepatic stellate cells by directly targeting MRP1/ABCC1. Oncol Rep.

[95] Konovalova J, Gerasymchuk D, Parkkinen I, Chmielarz P, Domanskyi A (2019). Interplay between MicroRNAs and oxidative stress in neurodegenerative diseases. Int J Mol Sci.

[96] Karnati HK, Panigrahi MK, Gutti RK, Greig NH, Tamargo IA (2015). miRNAs: Key players in neurodegenerative disorders and epilepsy. J Alzheimers Dis.

[97] Xiao J, Luo X, Lin H, Zhang Y, Lu Y, Wang N (2007). MicroRNA miR-133 represses HERG K+ channel expression contributing to QT prolongation in diabetic hearts. J Biol Chem.

[98] Zhao J, Zhou Y, Guo M, Yue D, Chen C, Liang G (2020). MicroRNA-7: Expression and function in brain physiological and pathological processes. Cell Biosci.

[99] Li L, Xu J, Wu M, Hu J (2018). Protective role of microRNA-221 in Parkinson’s disease. Bratisl Lek Listy.

